# Why Are Native Hawaiians Underrepresented in Hawai‘i's Older Adult Population? Exploring Social and Behavioral Factors of Longevity

**DOI:** 10.4061/2011/701232

**Published:** 2011-09-28

**Authors:** Lana Sue Ka‘opua, Kathryn L. Braun, Colette V. Browne, Noreen Mokuau, Chai-Bin Park

**Affiliations:** ^1^Myron B. Thompson School of Social Work, University of Hawai‘i, 1800 East-West Road, Honolulu, HI 96822, USA; ^2^Office of Public Health Studies, University of Hawai‘i, Honolulu, HI 96822, USA; ^3^Myron B. Thompson School of Social Work and Center on Aging, University of Hawai‘i, Honolulu, HI 96822, USA

## Abstract

Native Hawaiians comprise 24.3% of Hawai‘i's population, but only 12.6% of the state's older adults. Few published studies have compared health indicators across ethnicities for the state's older adult population or focused on disparities of Native Hawaiian elders. The current study examines data from two state surveillance programs, with attention to cause of death and social-behavioral factors relevant to elders. Findings reveal that Native Hawaiians have the largest years of productive life lost and the lowest life expectancy, when compared to the state's other major ethnic groups. Heart disease and cancer are leading causes of premature mortality. Native Hawaiian elders are more likely to report behavioral health risks such as smoking and obesity, live within/below 100–199% of the poverty level, and find cost a barrier to seeking care. Indicated is the need for affordable care across the lifespan and health services continuum. Future research might explain behavioral factors as influenced by social determinants, including historical trauma on Native Hawaiian longevity.

## 1. Introduction

Native Hawaiians are descendents of the aboriginal peoples inhabiting the Hawaiian archipelago prior to western contact in 1778 and exercising sovereign governance prior to the 1892 overthrown of the Hawaiian Kingdom by the United States (USA) [1, 2]. The 2000 US census enumerated 401,000 Americans (0.1% of the total population) of full or part-Hawaiian ethnicity, about 60% of whom reside in the State of Hawai‘i. Native Hawaiians comprise about 24.3% of the state's current population [3]. 

**Figure 1:**
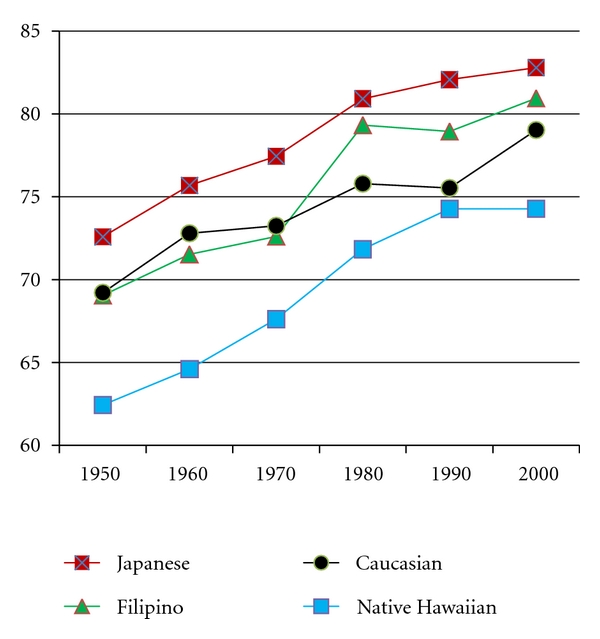
Life expectancy by ethnicity in Hawai‘i, 1950 through 2000.

As in other states, the population of Hawai‘i is aging, with an increasing number of residents living into old age. Although life expectancy in Hawai‘i exceeds that of other US states, studies conducted within the state reveal continuing ethnic differences in life expectancy [3]. As depicted in Figure 1, over six decades (1950–2000) Native Hawaiians have consistently had the lowest life expectancy when compared to the state's three other largest groups, namely, Caucasians, Filipinos (Americans), and Japanese (Americans). Notably, the magnitude of this disparity—about 10 years lower than the longest lived group—has not changed over time. About 16% of deaths among Native Hawaiians in 2005 occurred before 45 years, which is at least two times higher than for any other ethnic group living in the state [4]. Mortality disparities are particularly significant when comparing Native Hawaiians with Japanese; in 2005, 60% of deaths among Japanese occurred at age 80+ years, compared to only 25% of Native Hawaiians.

As a result, Native Hawaiians are underrepresented in the older age groups. In 2008, 21.4% of the state's overall population was 60+ years of age but only 11.1% of Native Hawaiians are in this age group. To look at it another way, Native Hawaiians comprised 24.3% of the total state population in 2008, but only 12.6% of residents age 60+ are Native Hawaiians. Table 1 displays population totals and age distributions of the state's four largest ethnic groups. 

Other investigators have examined data for the Native Hawaiian population in general and have found that Native Hawaiians have a higher prevalence of obesity, numerous chronic conditions, and greater impoverishment than other ethnic groups living in the state [5–7]. The relatively poor health status of the Native Hawaiian population overall has been of significant interest since the publication of the seminal* E Ola Mau: The Native Hawaiian Health Needs Study Report* [5]. Historic difficulty in Native Hawaiians' use of western mainstream healthcare services due to socioeconomic disadvantage, discrimination, and cultural misunderstanding are highlighted. Subsequently, social welfare researchers in gerontology and Native Hawaiian health have documented a number of socioeconomic disparities (e.g., higher rates of poverty, lower educational levels, homelessness, and incarceration) as well as health disparities (e.g., higher rates of diabetes and certain types of cancer, lower utilization of health services). To improve Native Hawaiian health and well-being, these researchers have underscored the need to consider more distal factors such as historical trauma or the cumulative effect of negative physical, sociocultural, political, and economic changes on current health disparities [7–12]. Concomitantly, they articulate the strengths of traditional Native Hawaiian culture, including those cultural values and practices on health, care giving, and social support that might be integrated into interventions which offer the prospect of increased longevity and enhanced quality of life [8, 10, 11].

Importance of this research notwithstanding, no recent studies have compared health indicators by ethnicity for older adults in Hawai‘i with a specific focus on Native Hawaiian elders [8]. Research described here attempts to address this gap in the current knowledge and was conducted by researchers associated with *Hā Kūpuna*: National Resource Center for Native Hawaiian Elders. Funded by the US Administration on Aging (AoA), *Hā Kūpuna *is one of three resource centers for native elders in the USA. The name “*Hā Kūpuna*” derives from the Native Hawaiian cultural belief that one's life essence (spiritual energy, ancestral knowledge) is transmitted to others through sharing of the *hā* (breath of life). It is believed that such sharing allows *kūpuna *(elders) to pass on vital knowledge and wisdom to subsequent generations, thus perpetuating valued cultural traditions and a positive sense of identity. Grounded in these and other traditional Native Hawaiian cultural values, *Hā Kūpuna* seeks to assure the transmission of *hā* from *kūpuna* (elders) to younger generations by achieving parity in life expectancy and good health among Native Hawaiian older adults comparable to that of other older Americans [13]. As a center dedicated to the health of Native Hawaiian elders, *Hā Kūpuna* develops and disseminates knowledge to inform policy and service innovations.

 In this paper, we examine proximal influences to the health and longevity of Native Hawaiian elders, specifically describe the causes of premature mortality among Native Hawaiians, and identify the ways in which sociodemographic and behavioral factors of Hawai‘i's elders vary by ethnicity. Our research was guided by these questions: (1) what are causes of premature mortality? (2) How does this vary by ethnicity? and (3) How do sociodemographic and health behavioral indicators vary by ethnicity? To address these questions we reviewed relevant statistical data collected by two Hawai‘i Department of Health surveillance programs.

## 2. Materials and Methods

Written requests for data were submitted to two surveillance programs of the Hawai‘i Department of Health (DOH): Vital Records and the Hawai‘i Behavioral Risk Factor Surveillance System (HBRFSS). Data sources are briefly described, as well as methods used to arrive at population-based statistics relevant for examining the underrepresentation of Native Hawaiian elders in the state's older (≥60 years) adult population. 

### 2.1. Causes of Premature Mortality

We requested data on the state's largest ethnic groups from the Vital Records program, which routinely gathers information on births, deaths, and marriages that take place in the state. Based on death record data, we calculated the Years of Productive Life Lost (YPLL) for the state's largest ethnic groups. YPLL is an index that measures the extent of premature mortality by giving a weight to each premature death from the predetermined cutoff age, with proportionally higher weights for younger deaths [14]. Any death before the cutoff age is defined as a premature death. Because YPLL is especially sensitive to the age distribution of the population, it cannot be used in cross-ethnic-group comparisons [15]. However, YPLL can be converted to a rate that is independent of sample size and population distribution following a method proposed by Lee [16]. These rates, herein called Total Population Life Lost per person in a lifetime (TPLL), can be compared across ethnic groups.

To construct TPLL, we obtained resident death data from Hawai‘i for the 3-year period, 1999–2001, for each age group by ethnicity, sex, and underlying cause of death of the deceased. Averaging 3 years of death data was done to smooth annual fluctuations in death. Cause of death is coded following the International Classification of Disease 10th Revision (ICD-10) [17]. We analyzed six causes of death common among older adults—cancer, heart disease, cerebrovascular disease, unintentional injuries, suicide, and diabetes—in addition to total death.

Both population and death data were classified into age groups, <1, 1–4 years, and then in 5-year intervals. All deaths in a given age group are assumed to have occurred at the midpoint age of the interval. We set age 70 as the cutoff for premature mortality in conformance with recommendations of the National Center for Health Statistics [18]. With the upper limit for the premature death at 70, the YPLL owing to premature mortality is given by

(1) $$\text{YPLL}=\sum \left(70-c_{i}\right)d_{i},$$

where, *c*_*i*_ is the midpoint of the *i*th age interval and *d*_*i*_ is the number of deaths in the *i*th age interval. Thus (70-*c*_*i*_) is the weight given to the deaths in the *i*th age group. The summation runs from age 0 to 70. For the cause-specific YPLL, *d*_*i*_ is the number of deaths from that particular cause of death. YPLL, as a function of d_i_, is to a large extent determined by the size and age distribution of the population as well as premature mortality. 

To convert YPLL to a rate, we applied the index proposed by Lee [16], which is independent of population size and age distribution. In Lee's method, the age-specific weight of YPLL is multiplied by the age-specific death rate of the population, that is,

(2) $$\sum \frac{\left(70-c_{j}\right)d_{j}}{p_{j}},$$

where *p*_*j*_ is the population of age *j*. The resultant is the annual *number* of YPLL expected to occur *per person* in age *j*. Originally, Lee named this measure as the cumulative rate of potential life lost (CRPLL). But here we call it the total potential life lost per person in lifetime (TPLL), since it also presents the number of premature deaths. When the age is grouped, TPLL is expressed by

(3) $$\text{TPLL}=\sum \frac{n{}_{i}\left(70-c_{i}\right)d_{i}}{p_{i}},$$

where *n*_*i*_ is the length of the interval of the age group. This is the total number of YPLL expected per person before age 70 in the study population. To establish 95% confidence intervals (CIs), we estimated the standard deviation of TPLL in each ethnic group

(4) $$\sum \frac{{\left[n_{i}\left(70-c_{i}\right)\right]}^{2}}{p_{i}}\frac{d_{i}}{p_{i}}\frac{p_{i}-d_{i}}{p_{i}}.$$

Further, as the 3-year average of death was used for *d*_*i*_, the variance of our TPLL is the above quantity divided by 3.

### 2.2. Variations in Socioeconomic, Clinical, and Behavioral Factors

We requested special data runs from the Hawai‘i Behavioral Risk Factor Surveillance System (HBRFSS) to examine ethnic variation in sociodemographic, clinical, and behavioral factors in the state's elders. Part of the Behavioral Risk Factor Surveillance System (BRFSS) of the Centers for Disease Control and Prevention (CDC), the HBRFSS gathers data by telephone from about 6,000 randomly selected adults (≥18 years of age). Respondents are queried about behaviors that directly or indirectly affect health and health-related topics. For example, BRFSS solicits information on height and weight to calculate body mass index (BMI), health behaviors (e.g., smoking), health screening (e.g., use of breast, cervical and colorectal cancer early detection screening), and chronic disease (e.g., diabetes, hypertension). Hawai‘i sample data are adjusted and weighted based on ethnicity distributions, and estimates are produced for Native Hawaiians, Caucasians, Filipinos, and Japanese. We requested a special tabulation that averaged responses from three years of data (2005, 2006, and 2007) relevant to older adults (≥60 years of age) residing in Hawai‘i. After years of data were combined, HBRFSS provided data tables for older adults as a whole, and for the four largest ethnic groups. The sample size of older adults over three years included 658 Native Hawaiians, 2,652 Caucasians, 561 Filipinos, and 1,840 Japanese, for a total of 6,346 elders.

## 3. Results and Discussion

### 3.1. Results: Causes of Premature Mortality

For Hawai‘i, the overall TPLL before age 70 per person from premature mortality was estimated at 3.3 years in 2000, but TPLL varied by ethnic group (Table 2). Among the groups we analyzed, the smallest number of years lost was observed for Japanese at 2.6 years and, as expected, the largest was for Native Hawaiians, at 5.3 years. The difference in TPLL between Japanese and Native Hawaiians is twofold, meaning that, on average, Native Hawaiians are losing twice as many years of potential life as Japanese. Among the six causes of death, cancer caused the largest number of potential years lost before age 70, with 0.74 years per person, accounting for more than 22% of overall TPLL. Heart disease, ranking second (0.59 years), accounted for 18% of overall TPLL. Unintentional injuries ranked third (0.40 years), accounting for 12% of overall TPLL. Death from suicide (0.21 years), stroke (0.13 years), and diabetes (0.06 years) ranked fourth, fifth, and sixth, respectively. 

**Figure 2:**
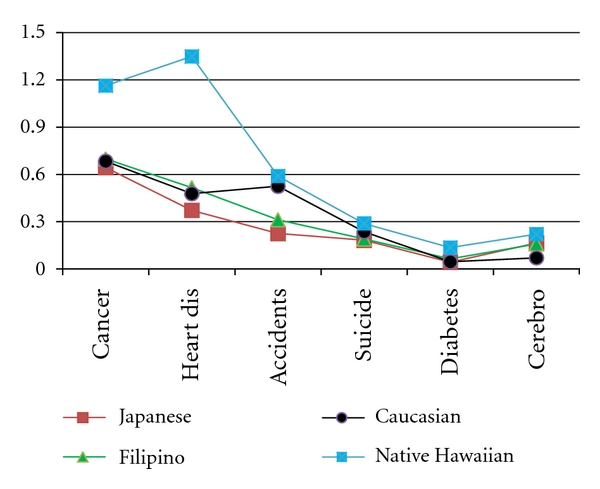
Total years of potential life loss by ethnic group and disease, 1999–2001.

As illustrated in Figure 2, the importance of cause of death varied considerably by ethnic group. Cancer was the most important cause of TPLL in all populations, except Native Hawaiians, while heart disease was top-ranked for Native Hawaiians. While cancer and heart disease were the top-leading causes of TPLL in most ethnic groups, accidents ranked second for Caucasians, while accidents ranked third for other groups. Suicide, cerebrovascular disease, and diabetes ranked fourth, fifth, and sixth in each ethnic group. Native Hawaiians had the highest TPLL for each of the six causes of death, almost twofold higher for cancer than the other groups, two-to-four times higher for heart disease, and two-to-three times higher for diabetes. 

### 3.2. Results: Sociodemographic and Behavioral Indicators

Sociodemographic data from HBRFSS indicate that Native Hawaiian older adults have the largest proportion of females to males; women account for 61.5% of Hawaiian elders compared to Caucasian females who account for 50.9% of Caucasian elders. This suggests that a disproportionate amount of Native Hawaiian men are dying before the age of 60 years. About 38.2% of Native Hawaiian elders attended at least one year of college. This percent is similar to that of Filipino elders (39.7%), but much lower than for Japanese (56.1%) and Caucasians (72.7%). Further, about 24% of Native Hawaiian elders are employed (perhaps indicating that they have had to delay retirement due to higher impoverishment rates), compared to only 18% of Japanese elders. 

According to HBRFSS, the percentage of elders with health insurance was relatively high overall (97%) although the percentage of Native Hawaiian elders reporting access to health insurance was 95%. Approximately 7.2% of Native Hawaiian elders reported not being able to see a physician in the past year due to cost, compared to less than 1% of Japanese elders. Native Hawaiian elders reported the highest prevalence of asthma (20% versus 11% overall) and diabetes (25% versus 17% overall). However, Native Hawaiians reported prevalence of heart attached, angina/CHD, and stroke similar to Caucasians. 

HBRFSS data indicate a relatively high prevalence of behaviors associated with increased disease risk among Native Hawaiian elders. For example, Native Hawaiian elders were most likely of the four ethnic groups to smoke every day or occasionally, about 14.6% compared to only 6.9% of Japanese elders (although Native Hawaiian elders with a history of smoking were most likely to state that they were trying to quit or have stopped smoking at least for one day in the past year). Native Hawaiian and Caucasian elders were most likely to report drinking more than four or five alcoholic beverages on one occasion (about 9% in each group). About one-third of all ethnic groups reported that they were overweight, however 36% of Native Hawaiian elders were obese compared to only 8% of Japanese elders. 

Data from HBRFSS indicate that 28% of Filipinos, 26% of Native Hawaiians, 23% of Japanese, and 19% of Caucasian elders rated their personal health as fair or poor. About 18% of Hawaiian elders responded that they were most likely to need special equipment, in comparison to 13% of all elders. About 80% of female elders reported having a mammogram within the last two years, including 78% of Native Hawaiian female elders. Among male elders, Native Hawaiians and Filipinos were the least likely to have had a Prostate-Specific Antigen (PSA) test for prostate cancer in the past 12 months (all male elders = 54%, Native Hawaiians = 37.7%, Filipinos = 33.7%). About 47% of Filipino elders and about 53% of Hawaiian elders have ever had a sigmoidoscopy or colonscopy to check for colorectal cancer, compared to 71% of Caucasians and 73% of Japanese.

## 4. Discussion

Data from two state surveillance programs highlight ethnic differences in cause of death, health, and behavioral indicators that may help to explain some of the ethnic differences in life expectancy. To summarize, the data demonstrate clear disadvantages for Native Hawaiian elders. For example, Native Hawaiian elders are least likely to have attended college and most likely to have incomes below 200% of poverty. They are least likely to have access to a Primary Care Physician, have routine checkups, or to participate in early detection cancer screening. Further, they are most likely to smoke and be obese. In comparison to other major ethnic groups, Native Hawaiian elders have the highest or second highest prevalence of a number of chronic diseases, including asthma and diabetes. Heart disease is the leading cause of premature death for Native Hawaiians, and Native Hawaiians lose significantly more years of productive life to heart disease, cancer, injuries, suicide, stroke, and diabetes than other ethnic groups. These findings for older adults are consistent with earlier research on the general Hawaiian population that documents serious health and social disparities [3–6, 9]. 

There were four major limitations to our research; these limitations point to a number of remaining and critical gaps in knowledge about Native Hawaiian elders. First, the study describes, but does not explain, the relationship of ethnicity and health indicators. Thus, caution must be exercised in using findings to directly inform policy or practice innovations. Continued research is needed to explain the relationship of Native Hawaiian ethnicity and proximal health indicators. Second, the study captured ethnic variation in health outcomes; the role of gender as it interacts with ethnicity was not explored. Research across the life course consistently exposes disparities by gender, and this is especially so in later life [19, 20]. For example, poverty rates for older women are nearly twice as high as for men. Our understanding of the lives of Native Hawaiian elders will be strengthened by the inclusion of gender in analyses of social and health disparities. Third, this study used surveillance data collected from Hawai‘i residents only; thus, findings cannot be generalized to populations outside the state, including Native Hawaiians living on the North American continent. At present, our knowledge of Hawaiian elders residing in the continental USA is very limited. This is an important omission since nearly 40% of Native Hawaiians live in the contiguous states, primarily California, Washington, and Oregon [21]. Extending life expectancy and improving quality of life requires that we understand life expectancies, health status, health care needs, preferences for care, and utilization patterns of all Native Hawaiian elders. Future research is needed to determine if Native Hawaiians on the continent experience similar disparities, such as shorter life expectancies than other ethnic groups in their new communities. 

Fourth and finally, the current study relied on surveillance data that did not address distal factors, such as the influence of historical trauma and systemic discrimination on current health disparities. Native Hawaiian and other indigenous health researchers consistently emphasize the influence of historical trauma and intergenerational marginalization on current health disparities [1, 5, 8–12, 22–26]. Sotero's Conceptual Model of Historical Trauma [25] posits that subjugation of a population by a dominant group has a cumulative effect on the physical, sociocultural, political, and economic well-being of the oppressed group. The trauma is felt by first-generation survivors (i.e., those who directly experienced the traumatic events), as well as by successive generations of their descendents. Across succeeding generations, the traumatic impact may be mitigated to some degree by cultural resiliency and other group protective factors. However, the result is an excess of social and physical ills that ultimately lead to population-specific health disparities. 

In the case of Native Hawaiians, historical records document the precipitous decline in population numbers and health status of Native Hawaiians following contact with the West [26]. At the earliest known point of contact in 1778, members of the Cooke exploratory expedition describe natives of the islands as hardy, robust, and capable of great physical activity. However, during the first 150 years of western contact, Native Hawaiians suffered disability and premature death from foreign diseases such as influenza, measles, small pox, syphilis, and mumps [1, 23]. Depopulation was severe; in the first century of contact the native population declined from an estimated 800,000 to 50,000 [26]. Depopulation was accompanied by cultural degradation with the native language and many traditional practices outlawed and/or subordinated as inferior to the English language and western practices, respectively [23, 24]. 

Among the most devastating changes were those related to shifts in the land tenure system from one of collective stewardship to one of private ownership and monopoly capitalism [24]. This shift assured the rise of westerner-owned plantations and eventually, led to the overthrow of the Hawaiian Kingdom in 1892 and US annexation in 1898. The exponential growth of the plantation economy and subsequent loss of Native Hawaiian sovereignty coupled with severe depopulation caused a collective grief among Native Hawaiian survivors [9, 23, 24]. *Na maka‘ainana* (those who tend the land, common people) were alienated from their *‘aina* (land), the source of their spiritual, social, and economic well-being. As natives lost access to their land, there was a mass exodus to port cities where they became wage laborers and in some cases, debtors, paupers, and *na pa‘ahao* (prisoners, convicts) [12, 24]. 

In line with Sotero [25], Native Hawaiian health researchers are linking the poor health of Native Hawaiians in contemporary times to this cascade of adverse historic events and intergenerational social marginalization [9, 10, 12]. Despite the relative success of Native Hawaiian organizations and groups to build cultural pride, positive identity, and holistic health in communities, the social marginalization of Native Hawaiians persists as reflected in social indicators spanning the breadth of the life cycle. For example, Native Hawaiian children are overrepresented in the state's child welfare population, Native Hawaiian youth are disproportionately represented in the state's juvenile justice system, and Native Hawaiian adult men and women are overrepresented at every stage of Hawai‘i's criminal justice system [2, 27]. 

The overall picture remains one of a population with more social and health disparities in comparison to the other large ethnic groups in the state. In the last few decades some social policies have been enacted to redress past wrongs and support programs aimed at reducing the disparate health outcomes affecting Native Hawaiians (e.g., Public Law 100–579, the Native Hawaiian Health Care Act of 1988). However, it is clear that more must be done to increase the longevity of Native Hawaiians and enhance the quality of life among Native Hawaiian elders. 

Our findings underscore the ongoing need to target behavioral risks affecting Native Hawaiian longevity across the life span. Health promotions grounded in evidence-based strategies and tailored on Native Hawaiian cultural preferences, as well as socio-economic circumstances have been recommended to reduce smoking and sedentary lifestyle, improve dietary practices, and increase participation in early detection screening [9–13]. In the past, health promotions that disregard relevant cultural, socio-economic factors, and systemic barriers have been received with distrust and even resentment by Native Hawaiian consumers who have experienced such efforts as cultural impositions [1, 12]. Thus, in targeting behavioral risks affecting longevity, it is crucial to develop interventions that adhere to principles of Community-Based Participatory Research [28] and meaningfully involve Native Hawaiian communities in identification of barriers and assets salient to intervention development and delivery.

## 5. Conclusions

Based on results from the current study, we conclude with three service and policy implications for Native Hawaiian elders. First, all Hawai‘i residents need primary health care that includes health education to promote optimal health practices, as well as chronic disease prevention and control. The inclusion of health promotion and disease prevention and control in primary care is especially important for Native Hawaiians who as a group have shorter life expectancies than other ethnic groups and who enter their senior years with more chronic conditions and poorer health habits than most other ethnic groups. Health promotion and disease management programs are needed to both inform Native Hawaiians of their risk for heart disease, and other chronic conditions, as well as to reduce the adverse impact of these conditions on their overall well-being. Contributors to heart disease, such as hypertension and high cholesterol, can be controlled by diet, exercise, and medications. Although many cancers cannot be prevented, they can be cured and/or controlled if diagnosed and treated early. Thus, enrollment in evidence-based health promotion and disease management programs should be encouraged financially and programmatically. 

Second, all Hawai‘i residents need health care to be affordable and this seems especially important for Native Hawaiian elders. Unfortunately, the high costs of deductibles, copayments, and noninsured treatments lead to delays in seeking necessary health care and may discourage elders from completing treatment or taking medications as recommended. Compounding the problem is the fragmented system of long-term care, both in the provision and funding of services. This issue has critical implications for Native Hawaiians, who may require long-term care services early in life due to earlier onset of disability. Nationally, long-term care services and financing are undergoing major programmatic changes because of the demand for cost-effective and efficient practices for improving quality of life of individuals in need of long-term care. Improvements to the long-term care system include Aging and Disability Resource Centers (ADRCs), which offer one-stop shopping for individuals in need of long-term care services, cash, and counseling programs (through which elders and caregivers are provided vouchers to pay for long-term care services and providers of their choice), expansion of community residential care models (such as assisted living, small group homes, and geriatric foster care), and the culture change movement (i.e., to make nursing homes more “homelike” and less institutional). The adaptation of these or other new models that aim to streamline and humanize long-term care, while reducing costs, should be balanced and sensitive to the health profile and needs of each* kūpuna* or Native Hawaiian elder. 

Third, services need to reflect older adult preferences [29]. For Native Hawaiian elders this may include preferences for culturally based programs and services. As all professionals will find themselves working with increasing numbers and proportions of diverse older adults with chronic disease, this becomes an increasingly important care component. Similar to all older adults, quality care for Native Hawaiian elders acknowledges their desire to remain in their own homes with an array of assistance from families, friends, and home and community-based services that honor and reflect their culture [9]. Not surprisingly, quality of life is influenced by the education and training of professionals and other service workers who provide the care. Successful interventions for Native Hawaiian elders are predicated on practitioners having an understanding of cultural values and traditions that influence elder's health practices, with cultural preferences that may include involvement of the elder's extended family and use of health promotion approaches reflecting holistic wellness [9–11, 13]. Continued effort is needed to develop an affordable and culturally responsive health care system that supports acute care, as well as health education and promotion, disease prevention, early disease management and treatment, and community-based long-term care. Further, services must be offered to people across the life span, thereby offering the opportunity for parity in life expectancy and the hopeful prospect of good health to Native Hawaiian elders comparable to that of other older adults.

##  Disclosure

The information in this paper does not reflect the option of the US Administration on Aging.

## Figures and Tables

**Table 1:** Population totals and distributions of young people, adults, older adults by four largest ethnic groups, Hawaii, 2008.

	Native Hawaiian	Caucasian	Filipino	Japanese	Total
	305,838	259,851	200,042	273,016	1,257,607
	24.3%	20.7%	15.9%	21.7%	100%
column %					
<20 years	38.0%	13.1%	29.2%	17.2%	25.4%
Age 20–59	50.9%	55.3%	55.1%	51.0%	53.3%
Age 60+	11.1%	31.6%	15.7%	31.8%	21.4%%

Total	100.0%	100.0%	100.0%	100.0%	100.0%%

row %					
<20 years	36.4%	10.6%	18.3%	14.7%	100.0%
Age 20–59	23.3%	21.5%	16.4%	20.8%	100.0%
Age 60+	12.6%	30.6%	11.7%	32.3%	100.0%

**Table 2:** Years of Total Potential Life Lost (TPLL) and standard errors for the total population and 4 ethnic groups by cause of death, 1991–2001.

Cause	Native Hawaiian	Caucasian	Filipino	Japanese	Total population
All Causes	5.28 (0.12)	3.38 (0.10)	3.03 (0.11)	2.57 (0.11)	3.30 (0.05)
Cancer	1.16 (0.05)	0.68 (0.03)	0.70 (0.04)	0.64 (0.04)	0.74 (0.02)
Heart Disease	1.35 (0.06)	0.48 (0.03)	0.52 (0.04)	0.37(0.03)	0.59 (0.02)
Injuries	0.59 (0.05)	0.52 (0.04)	0.31 (0,04)	0.22 (0.04)	0.40 (0.02)
Suicide	0.29 (0.03)	0.24 (0.03)	0.19 (0.03)	0.18 (0.03)	0.21 (0.01)
Stroke	0.22 (0.02)	0.07 (0.01)	0.15 (0.02)	0.16 (0.02)	0.13 (0.01)
Diabetes	0.16 (0.02)	0.05 (0.01)	0.06 (0.01)	0.04 (0.01)	0.06 (0.01)

**Table 3:** Health indicators in 60+ population by ethnicity, HBRFSS, 2005–07.

		All people age 60+	Native Hawaiian	Caucasian	Filipino	Japanese
Self-rated helath, insurance, checkups, MD visits	Rate health as fair/poor	22.7	26.3	19.0	28.0	22.5
	Need adaptive equipment	12.8	18.0	12.8	14.0	11.2
	Health insurance	97.2%	95.3%	96.8%	97.5%	98.4%
	Could not see MD in past 12 months due to cost	3.2%	7.2%	3.8%	3.8%	0.6%
	No PCP	4.9%	6.3%	7.0%	3.8%	2.8%
	Routine checkup in past year	82.9%	81.0%	79.4%	81.3%	86.4%

Chronic conditions	Told by MD with asthma	10.8%	19.7%	11.8%	10.2%	7.6%
	Told by MD have diabetes	16.5%	24.9%	11.9%	23.8%	14.9%
	Told by MD had heart attack	9.6%	11.3%	12.0%	8.7%	7.2%
	Told by MD have angina/CHD	8.6%	11.4%	10.1%	6.4%	6.5%
	Told by MD had stroke	6.8%	7.2%	7.5%	4.4%	6.7%

Lifestyle risks	Smoked 100+ cigarette in lifetime	48.1%	56.8%	57.0%	37.2%	43.1%
	Current smokers (daily/occasional)	8.9%	14.6%	9.8%	9.7%	6.9%
	Binge drinkers (4-5 + drinks at once)	7.0%	8.9%	9.0%	4.0%	5.9%
	Obese	16.5%	36.2%	19.2%	16.1%	7.7%
	Participated in physical activity outside of regular job in past 30 days	77.0%	72.9%	81.4%	72.1%	75.6%

Screening practices	For women, had mammogram in last 2 years	81.3%	77.7%	76.2%	82.3%	85.8%
	For men, had PSA test in past 12 months	54.0%	37.7%	58.8%	33.7%	60.5%
	For men, had DRE in past 12 months	45.0%	34.2%	48.6%	28.2%	49.7%
	Ever had sigmoidoscopy or colonoscopy	66.0%	52.7%	70.6%	47.3%	73.0%
